# Deep brain stimulation of the subthalamic nucleus modulates sensitivity to decision outcome value in Parkinson’s disease

**DOI:** 10.1038/srep32509

**Published:** 2016-09-14

**Authors:** Ben Seymour, Michael Barbe, Peter Dayan, Tamara Shiner, Ray Dolan, Gereon R. Fink

**Affiliations:** 1 Computational and Biological Learning Lab, Department of Engineering, University of Cambridge, UK; 2 Wellcome Trust Centre for Neuroimaging, UCL, London, UK; 3 Center for Information and Neural Networks, National Institute for Information and Communications Technology, Japan; 4 Department of Neurology, University Hospital, Cologne, Germany; 5 Cognitive Neuroscience, Institute of Neuroscience and Medicine (INM-3), Research Centre, Jülich, Germany; 6 Gatsby Computational Neuroscience Unit, UCL, London, UK

## Abstract

Deep brain stimulation (DBS) of the subthalamic nucleus in Parkinson’s disease is known to cause a subtle but important adverse impact on behaviour, with impulsivity its most widely reported manifestation. However, precisely which computational components of the decision process are modulated is not fully understood. Here we probe a number of distinct subprocesses, including temporal discount, outcome utility, instrumental learning rate, instrumental outcome sensitivity, reward-loss trade-offs, and perseveration. We tested 22 Parkinson’s Disease patients both *on* and *off* subthalamic nucleus deep brain stimulation (STN-DBS), while they performed an instrumental learning task involving financial rewards and losses, and an inter-temporal choice task for financial rewards. We found that instrumental learning performance was significantly worse following stimulation, due to modulation of instrumental outcome sensitivity. Specifically, patients became less sensitive to decision values for both rewards and losses, but without any change to the learning rate or reward-loss trade-offs. However, we found no evidence that DBS modulated different components of temporal impulsivity. In conclusion, our results implicate the subthalamic nucleus in a modulation of outcome value in experience-based learning and decision-making in Parkinson’s disease, suggesting a more pervasive role of the subthalamic nucleus in the control of human decision-making than previously thought.

Treatment of Parkinson’s disease (PD) with subthalamic nucleus (STN) deep brain stimulation (DBS) is generally thought to disrupt (more precisely, to interfere with abnormal synchronization of) the STN^1^,^2^. DBS can yield subtle but complex behavioural changes, extending beyond that attributable to reductions in dopaminergic medication following alleviation of motor symptoms^3^. Accruing data indicate important changes in patients’ decision-making behaviour, with impulsivity being the most commonly documented manifestation^4^,^5^,^6^. Taking note of the fact that the unstimulated STN may not be functioning normally in PD, it suggests that the STN subserves a fundamental role in modulating decision-making. However, the precise nature of this function (or functions) remains unclear.

There are two broad perspectives concerning the role of the STN in decision-making. The first focuses on response inhibition, in which the STN may convey a global stop-signal or ‘set of brakes’ to inhibit pre-potent responses^7^. This is manifest, for instance, in premature task responding seen in STN-lesioned animals^8^,^9^, and enhanced BOLD responses associated with response inhibition in human neuroimaging studies^10^,^11^. Response inhibition also potentially underlies response switching, when an optimal response requires inhibiting a sub-optimal one^12^,^13^.

The second class focuses on inhibition of early responding in the face of decision conflict^14^,^15^,^16^,^17^,^18^. These draw on a mechanistic (computational) account of the decision process which invokes a dynamic competition between two (or more) competing alternatives, formalized for example within race and drift diffusion models^19^. Accordingly, the STN is thought to set a decision threshold that determines when enough evidence has accrued to allow a response. Impulsivity in this situation involves premature and more error-prone responding.

However, it seems unlikely that these accounts explain all aspects of the behavioural phenotype of STN-DBS. In particular, beyond the sequel of premature responding, it is unknown whether fundamental choice-determining components of the decision-process, such as value, learning, and time, depend on the STN. For example, a recent study of PD patients showed that STN-DBS induces overestimation of own performance, increased risk-taking and preference for competitive environments^20^. To investigate this further, we studied the performance of Parkinson’s disease patients whilst ON and OFF STN DBS in two decision-making tasks designed to probe distinct aspects of impulsive choice. We adopted a computational approach to the analyses, intended to probe beneath global performance and thereby to interrogate functional mechanisms whence this behaviour emanates. We fit computational models to behaviour to isolate distinct components of the choice process. Our results highlight a specific role for the STN in learned outcome value, in the absence of any observable effects on inter-temporal choice, learning rate, reward-loss competition, or perseveration.

## Materials and Methods

### Subjects

We recruited 22 adult patients (mean age 55 yrs, standard deviation 7.5 yrs, 18 male, 4 female) diagnosed with Parkinson’s disease according to the British Brain Bank criteria^21^ at the Klinik und Poliklinik für Neurologie in Cologne, Germany. Patients between Hoehn and Yahr stages I–III were included. Patients had received implantation of electrodes into the subthalamic nucleus for deep brain stimulation on average 8 years after initial diagnosis. For implantation, the dorsolateral STN had been assessed visually by preoperative stereotactic magnetic resonance imaging and computed tomography, as in standard clinical practice. Stereotactic X-ray was used for postoperative lead localisation. Patients were tested at least 3 months after implantation (the median interval between surgery and participation in the study was 333 days; range 91 days to 5 years) so that microlesional effects could be excluded. The individual stimulation parameters of each patient were used in the ON stimulation condition. All subjects gave written consent, and the study was approved by the local ethics committee (Ethikkommission der Medizinischen Fakultät der Universität zu Köln). The study was carried out in accordance with approved guidelines.

### Design and setting

Subjects performed both tasks whilst ON and OFF DBS in a within-subject design. This was carried out on the morning and afternoon of the same day, in randomised order (to provide plenty of ‘washout’ time). Prior to OFF testing, STN-DBS was paused for one hour. In addition, subjects attended the clinic after cessation of all dopaminergic medication for at least 12 hours, so that both ON and OFF manipulations were implemented without medication. To look for evidence of impulsivity, we employed the Barrett Impulsivity Scale, a trait level index of symptomatic impulsivity previously shown to detect impulsivity in Parkinson’s disease^22^,^23^.

### Task details

#### Experiment 1: Instrumental learning of rewards and punishments

**Figure 1:**
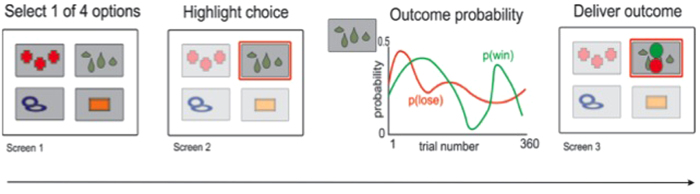
Instrumental learning task design. Subjects were required to pick one of four options on each trial. 3 seconds after selection, the option yielded an outcome that could comprise reward (token for €1) and/or punishment (token for -€1). The figure shows an example of combined reward and punishment outcome. The probabilities of reward and punishment were fully independent from each other, independent between options, and varied slowly over trials as indicated in the exemplar graph for one option. Following the outcome there was an inter-trial interval of 3 secs.

We employed a ‘4-armed bandit’ instrumental decision-making task, similar to one we previously employed in healthy individuals^20^. The task involved repeated choices amongst 4 options to try and win tokens yielding money and simultaneously, but independently, avoid accruing tokens signalling loss of money. On each trial, subjects could choose 1 of 4 options, signified by abstract pictures on a computer screen, each of which had a probability of yielding a positive (green) token, and a different probability of yielding a negative (red) token (Fig. 1). Subjects could receive green, red, neither or both tokens on each trial. At the end of the task, subjects exchanged the sum of the positive tokens minus the sum of the negative tokens for money, with a net value of +/− €1 per token.

Several important features of the task enabled us to look separately at behaviour influenced by reward and punishment, and to disambiguate learning and performance. First, the probabilities of winning or losing tokens were independent of each other, and independent between each option, such that subjects needed to learn about the distinct reward and loss information of each option individually. That is, for each option, the probability that the option would return a reward was independent of the probability it would return a loss, and so knowing the probability of reward would tell you nothing about the probability of loss, and vice versa. Second, the probabilities of reward and loss varied slowly over time for each option (according to Gaussian random diffusions), requiring subjects constantly to balance exploration (information accrual^24^) and exploitation (winning and avoid losing money). That is, one option might begin as good, and slowly become bad over time, and other options might do the opposite, or they might change little. Hence behaviour on the task involves two trade-offs: balancing reward-based and punishment-based information, and balancing information exploration and exploitation. Third, the outcome probabilities of any option was independent of other options. That is, there was no relationship between the different options – knowing that one option was good (frequently yielded reward and infrequently yielded loss) would tell you nothing about the other options – they could all be good, or bad, or anything. This independence of probabilities both between reward and loss, and between options, removes any higher-order structure from the task: i.e. no reciprocal inference can be made about loss probability from reward probability. Therefore if an option paid frequent reward tokens, for example, it does not necessarily mean it pays fewer loss tokens. Similarly, if a subject has found a profitable option, this does not mean that other options are necessarily less profitable. Such higher-order structure frequently confounds interpretation of performance in decision-making tasks, because it is independently learnable.

Patients performed 360 trials in total, with the 4 options being displayed in the same position on each trial. If no choice was made after 10 seconds (which across subjects occurred either never, or very rarely), the next trial was presented automatically. Otherwise, there was no requirement to respond quickly on the task, and subjects were aware of this from the beginning. After a choice was made, all options remained on the screen with the chosen option highlighted, and an interval of 3 seconds elapsed before presentation of the outcome. If the subject won the reward, a ‘Reward’ token appeared overlaid on the chosen option image. If the subject received a loss, a ‘Loss’ token appeared overlaid on the chosen option. If both loss and reward were received, both tokens simultaneously appeared overlaid on the chosen option, one above the other. The outcome was displayed for 1 second, after which the bandit was extinguished and the screen was blank for 3 seconds.

Subjects were instructed before the experiment that all ‘Reward’ tokens would be exchanged for €1, and all ‘Loss’ tokens would incur a cost of €1, and that the net amount would be faithfully remunerated. The exact amount was paid by cash transfer to the subjects after the experiment, along with a small fixed reimbursement for time and inconvenience. The rationale for using tokens in the actual task rather than money was to allow separate currencies for reward and loss, which were independent during the study (and to allow for convenient simultaneous delivery of reward and loss outcomes).

### Data Analysis

We first performed a standard frequentist analysis of total reward and loss accrual between DBS conditions, using paired t-contrasts. Next, we looked at the frequencies of switching or not switching after different outcomes, to give a basic metric of the immediate influence of different outcomes on choice. We then performed a computational reinforcement learning analysis, identical to that previously described^24^. The advantage of this over a switch/non-switch analysis is that it looks for acquisition of value over time, beyond the last trial alone. Specifically, reinforcement learning describes the general algorithmic process by which humans (and animals) learn from experience^25^, and is a computational rendition of Thorndike’s Law of Effect^26^. It formalizes the fact that rewards tend to reinforce an option, increasing the probability it will be chosen again on a subsequent trial. Most reinforcement learning models work by calculating a running average of the value of different options – updating this value with the outcome of each new option according to a learning rate. A high learning rate that the value is very sensitive to the latest outcomes, and less sensitive to much earlier outcomes. This can also be thought of as reflecting an influence that declines exponentially with time, such that recent outcomes influence decisions more than distant ones. In this sense, a high learning rate is equivalent to a high exponential decay rate. Similarly, losses are expected to decrease the probability of the option concerned. More formally, we can specify the model as follows: the net reward weight 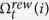 for option *i* on trial *t* is given by the history of rewards in the recent past:


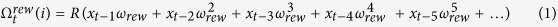


where 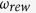
specifies the decay weight (i.e declines exponentially in the successively more distant past, equivalent to the learning rate in reinforcement learning as applied to chosen and non-chosen actions on each trial), 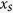 is 1 if a reward was delivered at time *s*, and 0 otherwise, and R is a measure of the sensitivity of the subject to the reward, which is multiplied by the expression in brackets. The net punishment weight 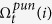 is determined similarly, given the recent history of punishments:


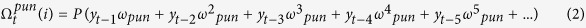


where 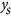 is 1 if pain was delivered at time *s*, and 0 otherwise. P is the punishment sensitivity (multiplied by the expression in brackets).

We also incorporate an inherent tendency to repeat the last choice irrespective of outcomes (i.e. ‘pure’ perseveration) using a choice bias weight *C*_*i*_^27^. This is positive for a ‘perseverative’ tendency to repeat choices, and negative for an ‘anti-perseverative’ tendency to switch choices.

The overall tendency to choose a particular option is then determined by a logistic choice (softmax) rule based on the sum of these independent weights:


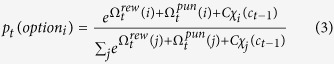


where *c*_*t-1*_ is the actual choice of the subject on trial *t-1* and 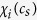 is 1 if option *i* was chosen on trial *s* and 0 otherwise. Note that there is no ‘temperature’ parameter, as this is subsumed within the reward/punishment weights. As before, we use a maximum likelihood method to determine the parameters given the data:


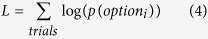


The reinforcement learning approach can be seen as a form of conditional logit regression analysis, in which the weights according to which preceding outcomes influence choice are constrained to decay exponentially.

For assessment of response times and conflict, as in the discounting analysis, we used the model-based analysis to define difficulty in terms of a difference in estimated value, on a trial-by-trial basis, between the highest two valued options. Easy and hard trials were again defined as those with differences in value that were greater or less than the median, respectively.

#### Experiment 2: Temporal Discounting

Patients performed an inter-temporal choice task, based on one we previously designed^28^,^29^. The task involves choosing which of two financial options they would prefer to receive. Each option involved an amount of money (between €1–€100) to be paid at a given delay (between 3 to 48 weeks). This typically forces the subject to commit to a choice between smaller sooner amounts of money, and larger more distant amounts. People who favour the smaller sooner option are considered to be more temporally impulsive.

**Figure 2:**
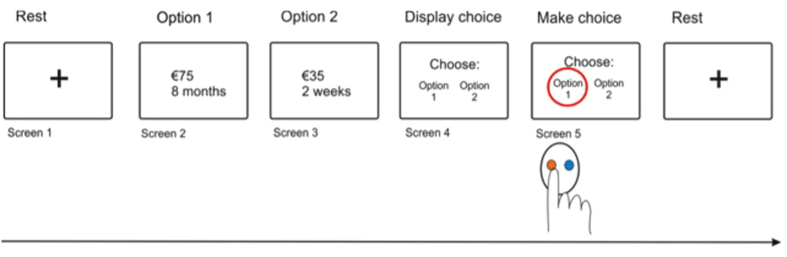
Temporal discounting task design. On each trial, patients were presented with two financial options, in sequence. Each option consisted of an amount of money and a delay. After both options had been presented (for 3 seconds each), subjects made their choice with a button key-pad, and moved on to the next trial after a short period. Patients knew that at the end of the task, their chosen option from one randomly-picked trial would be paid faithfully.

On each of 220 trials, the two options were presented sequentially on a computer monitor, for 3 seconds each (Fig. 2). Patients then selected the option they would prefer using a button press, after which their choice was highlighted for 3 seconds, followed by an inter-trial interval of 3 seconds. There was no requirement to respond quickly, and patients were informed of this. Patients made their choices with the knowledge that one of their choices would subsequently be selected at random, and that this choice would be remunerated exactly according to the amount of money involved and delay specified. This was faithfully carried out, involving a random selection of one of the trials, and subjects receiving the money via bank transfer on the nearest working day to their chosen option. This approach which entails fulfilling subjects’ choices for real is standard practice in behavioural economics, and encourages subjects to make choices according to their true preferences. We therefore aimed for ecologically valid amounts of money and delays. Subjects received the same 220 options in the task when ON and when OFF, to allow accurate comparison between conditions. The specific delays and amounts used for the two options were selected to lie roughly in the region of indifference, based on our previous data from healthy volunteers^28^,^29^.

### Data Analysis

The analysis follows that of our previous studies in healthy individuals^28^,^29^. We first compared the number of times patients chose the sooner amount over the larger amount, using a sign test. To study the distinct components of choice, we adopted a computational approach based on current neuro-economic models of the functional basis of inter-temporal choice^30^,^31^. Specifically, such models propose that choice depends on two critical processes. First, subjects need to evaluate how much the worth of money is discounted by the given delay. Current theories assume that discounting is a hyperbolically decreasing function^32^,^33^ (but see ref. 34) such that subjects who discount more rapidly are more temporally impulsive. Second, subjects need to evaluate the utility of the amount of money, given the fact that utility is typically not a linear function of amount. This is captured by the principle of decreasing marginal returns, in which successively greater increments of money yield marginally smaller additions to utility^35^. As an illustration, the marginal increment in utility arising from addition of €10 to a €20 option is greater than that associated with addition of €10 to a €200 option.

We formalised each of these processes, thus parameterising the extent to which individual subjects discount time and utility. We then compared these parameter estimates between DBS ON and OFF conditions. There are slightly different ways of computationally specifying temporal and utility discounting; therefore, we first compared the likelihoods of a number of related models, and selected the best fitting model. As in Pine *et al*.^29^, we compared the following models:

Hyperbolic discounting with linear utility


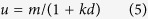


Exponential discounting with linear utility


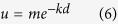


Hyperbolic discounting with power utility


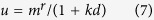


Hyperbolic discounting with inverse exponential utility


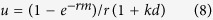


Exponential discounting with power utility


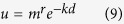


Exponential discounting with inverse exponential utility


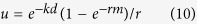


In the above equations, *u* is the utility of the option, where *m* is the amount of money, *d* is delay in time. The parameters *k* represents a time discounting parameter, and the parameter *r* represents a money discounting parameter. Both simply parameterise the discounting effects according to the non-linear function specified in each of the equations. These equations allow us to calculate the utility of each option. We also formalised the decision rule by which the two options (*i* and *j*) yield a choice probability. Here, we used a standard logistic decision rule:


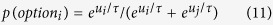


Here, as is conventional in human/animal decision-making, formal models of choice are generally stochastic, in that they determine the probability of a choice given any set of option utilities^36^. Such a stochastic process quantifies the ‘noisiness’ of each decision, parameterised by *τ*, and provides a non-specific index of performance consistency. Thus to calculate the likelihood of each model, we computed the sum of the log likelihoods over all trials:


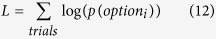


We estimated the overall likelihood of each model, with the most likely parameters (*k, r, τ)* for each model (fit on an individual subject basis, to provide point estimates of individual parameters), by minimising the likelihood function (using standard minimisation functions implemented with Matlab (Mathworks, Inc)), and selected the best model using a Bayesian Model Comparison procedure using the Bayeisan Information Criteria. This incorporates a standard penalty for model complexity (number of parameters).

To assess whether response times were modulated as a function of difficulty, we first ‘normalised’ the reaction time data, and then used our model of discounted, non-linear utility, to define difficulty. Normalization was based on fitting a gamma distribution to the response time data; this captures the characteristic distribution of response times (separately parameterised when ON and OFF). We then used the corresponding gamma probability density function to generate the corresponding normalised response time values. Next, the difficulty of a trial was defined in terms of the difference between the values of the two options on that trial, estimated by the best-fitting behavioural model. In particular, easy and hard trials were defined as those whose value differences were greater and less (respectively) than the median value difference.

It is worth noting that the clinical OFF state might incorporate other unexpected confounds in studies of PD, such as effects on cognitive load and effort that could induce different cognitive strategies. These could be pervasive across many similar studies, but are extremely hard to evaluate and control for.

## Results

On clinical assessment, patients experienced significant improvement in clinical signs of their Parkinson’s disease when stimulation was on (DBS ON: Unified Parkinson’s Disease Rating Scale (UPDRS) = 16.91/108) compared to off (DBS OFF: UPDRS = 35.55/108). The mean impulsivity index measured (whilst ON) using the Barrett Impulsivity scale (BIS) scale was 60.6 (s.d. = 10.0).

### Instrumental reward-loss learning task

Patients performed significantly worse when ON than when OFF, with mean net winnings of €7.82 and €15.50, respectively (sign test p < 0.05)(Table 1).

To examine this further, we looked at the frequency of choice repetition (i.e. sticking with the previously chosen option, versus switching to a different option) with a particular option, following different outcomes. There was no difference between the *overall* frequencies of sticking/switching with/from an option between groups. However, DBS OFF patients were significantly more likely to switch after punishment (p < 0.005), and had a marginal trend to stick more often after rewards (p = 0.08) (see Table 2). These results suggest that subjects were more sensitive to outcomes (at least, punishments) when OFF than ON. We found no correlation between impulsivity, as measured by the BIS score, and either marginal improvement in overall performance, or tendency to stick/switch.

**Figure 3:**
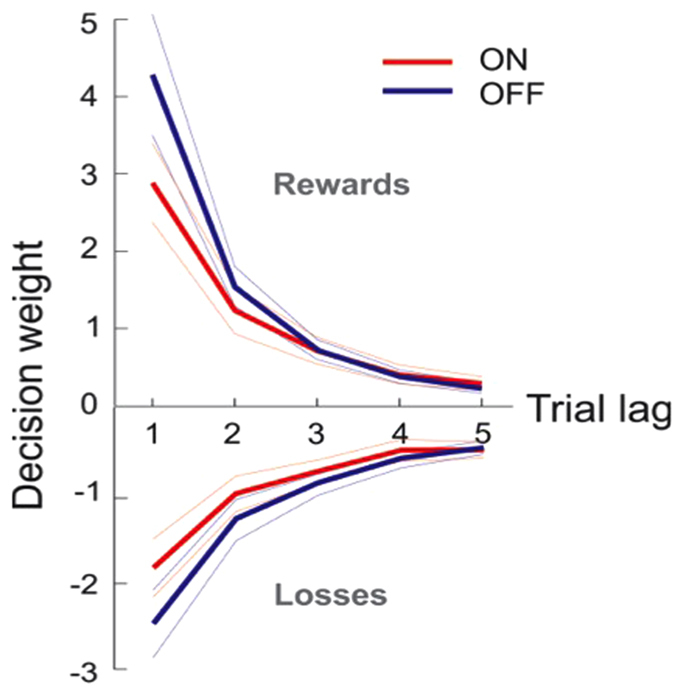
Instrumental performance between conditions. Decision weights are shown at successive trial lags for rewards and losses, when subjects are ON (red) and OFF (blue) stimulation. The initial magnitude of the weight (at Trial lag = 1) reflects the outcome sensitivity, and is significantly reduced in the DBS ON group. The decay rate over successive trial lags reflects the learning rate, and is not significantly different between groups.

To examine performance in more detail, we fit a reinforcement learning model to the behaviour. This estimates the impact of rewards and losses on subsequent choices, extending the influence of outcomes on all subsequent choices over time, and not just immediately succeeding choices (as a simple switch/stay analysis does). By capturing the core components of the learning process, such models are much more sensitive to identifying precise effects. The model involves two key parameters each for rewards and losses: first, the sensitivity to the outcome represents the *magnitude* of the influence of outcomes in terms of their ability to modify subsequent choices: second, is a learning/forgetting rate, which effectively determines how sensitive subjects are to changes in contingencies with low rates associated with a slowly adaptive but persistent influence over trials (we also incorporated an additional parameter that estimates an inherent tendency to repeat options irrespective of outcomes, i.e. perseveration - see methods). The influence of rewards and punishments can be easily represented graphically, which intuitively illustrates the nature of reinforcement learning (Fig. 3).

As shown in Fig. 3, we found a significant reduction in both sensitivity to rewards and losses when DBS was ON. There was no differential reduction between reward and loss sensitivity, i.e. no change in the effective reward-loss trade-off. The parameters are reported in Table 3. In contrast, we found no difference in either the learning rates or perseveration between the conditions.

We found that patients responded significantly faster when ON than OFF (mean response time 676.2 ms (ON) and 768.5 ms (OFF)). To look at impulsivity associated with decision conflict, we classified trials as easy or hard according to the difference in values of the best and second best options on each trial, estimated within the model-based analysis. In both ON and OFF conditions, after normalising the response times to allow accurate comparison across subjects (see methods) we found that patients responded slightly more slowly for more difficult trials. However, the interaction (condition x conflict difficulty) did not reach significance (p = 0.08 paired 2-tailed post-hoc t-test; see Table 4). We also studied the proportion of correct choices (defined as the selection of the highest value from the model-based analysis) in the easy and hard trials, to see whether ON subjects’ worse performance related specifically to difficult choices. This was not the case: ON subjects selected the correct bandit on 86.2% and 60.3% of easy and hard trials respectively, compared to 89.3% and 58.8% when OFF.

In summary, the predominant finding was a specific reduction of outcome sensitivity to both rewards and punishments, that can’t be easily explained by a change in learning (per se), reward-loss trade-off, or decision conflict.

#### Inter-temporal choice task

Subjects made choices between amounts of money between €1 and €100 euro, to be remunerated at delays between 3 and 48 weeks, fully aware that one of these choices would be randomly selected and paid according to its description. The vast majority of trials involved choice between a sooner option involving a smaller amount than a later option. Over 220 trials, repeated when subjects were DBS ON and DBS OFF, we first compared the frequency with which they chose the sooner option. This revealed no difference between conditions: when ON, the mean frequency for choosing the sooner was 120.5 (SEM 13.7), and when OFF, the mean frequency was 117.1 (SEM 12.9) (2 tailed t-test p = 0.3). Thus, there was no global effect of DBS on performance. Furthermore, there was no effect on response time (mean 5.85 secs (ON) and 5.67 secs (OFF)).

Global performance can mask differential effects on individual components of valuation in inter-temporal choice tasks. This is the case since impulsivity can be manifest either by time-preferences (the relative utility given to a fixed amount of money over increasing delays), and also by magnitude-preferences (the relative utility given to increasingly larger amounts of money)^37^. This latter effect is a characteristic of the fact that utility functions for money are typically concave, an observation known as the principle of ‘decreasing marginal returns’ (see methods). In such cases, a more concave utility function yields more impulsive choices. In principle, therefore, it is possible for opposite effects of time and magnitude to exist in the absence of significant changes in global performance measures on discounting tasks.

To address this latter possibility we adopted a model-based approach, designed to parameterise the distinct components involved in inter-temporal choice according to a computational model. We first calculated the log likelihood of six similar, competing models, and performed Bayesian model comparison to select the most likely model (see methods). As previously reported^37^, discounting models that incorporated a concave utility function provided the best fit to the data.

The model comparison is shown below (Table 5), where it can be seen that a hyperbolic model with power utility function provides the best fit to the data. Therefore, this model was used to calculate the parameters for the remainder of the analysis. These parameters were then taken as a summary statistic and compared between conditions using t-contrasts.

**Figure 4:**
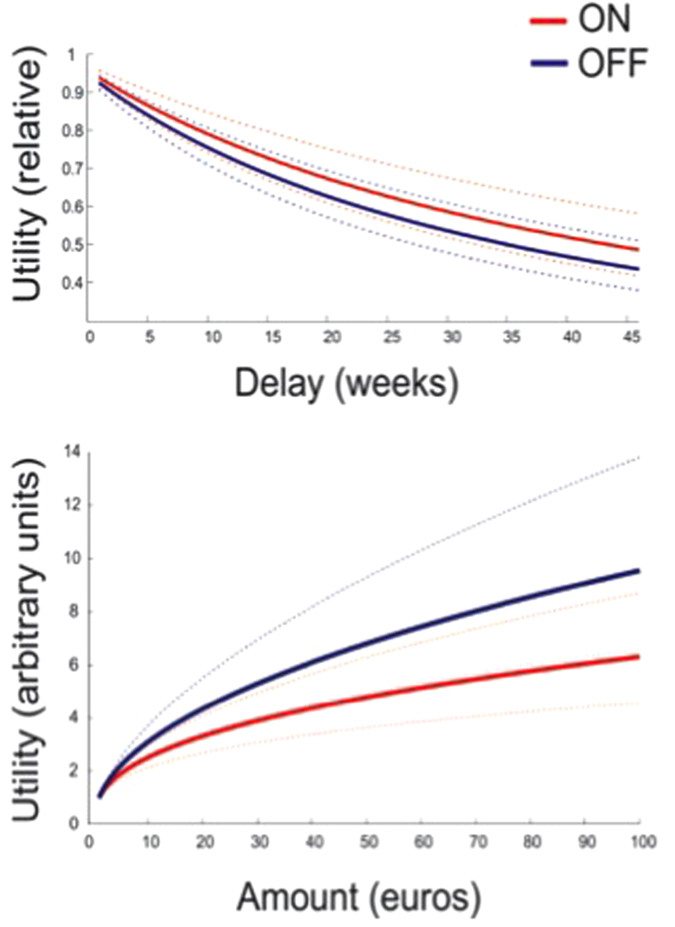
Results – inter-temporal choice. Estimated delay and magnitude discounting functions in patients whilst on (red) and off (blue). In the case of delay, the curves represent a hyperbolically decreasing function in which more distant outcomes are discounting. In the case of magnitudes, the increasing amounts result in decreasing marginal returns, manifest as a concave utility function. The dotted lines represent +/− 1 standard error of the mean. There is no statistical difference between on and off stimulation in either case.

The computational model incorporates 3 key parameters: a time discounting parameter (‘k’, from a hyperbolic function), a concave utility parameter (‘r’, from a power utility function), and a parameter that determines the consistency of choice (‘β’, from a logistic sigmoid choice rule). We estimated these parameters from each patients’ trial-by-trial choices when ON and OFF stimulation, as shown in Table 6. This showed no evidence of any differential effects of time- and utility- preferences. Figure 4A shows the estimated time and utility preferences for the ON and OFF conditions.

We did, however, observe a significant, albeit small, difference in the temperature (*τ*) parameter (p < 0.05 two-tailed t-test, uncorrected for multiple comparisons), which suggests a mild, non-specific performance improvement when subjects were ON stimulation. That is, subjects showed a slightly more deterministic (consistent) behaviour when ON, but with no significant change in their choice preferences. However, since this was result was on just significant at an uncorrected (for multiple comparisons) threshold of p < 0.05, it should be considered exploratory.

We next considered whether patients with higher BIS impulsivity ratings (a measure of trait mpulsivity) might be more prone to DBS-induced temporal impulsivity. We therefore correlated the BIS score with the marginal difference in selection of the sooner option between the ON and OFF sessions. However, there was no significant correlation (r = 0.38, p = 0.08 paired, two-tailed t-test, Fig. 4B).

Finally, even though the task did not involve any requirement to respond quickly, we considered whether subjects manifest impulsivity as increased response latencies with high conflict choices^15^. High conflict was specified as trials in which the difference between the estimated values was less than the median value (given the computational model, using the subjects’ individually estimated parameters). We found that both ON and OFF stimulation, patients manifest a slight (paradoxical) response speeding, i.e. lower response latencies for higher conflict trials (Table 7). However there was no main effect of stimulation and no interaction between condition and conflict.

In summary, we found no convincing evidence of significant changes in temporal discounting in STN ON and OFF states.

## Discussion

Our aim was to probe whether STN DBS modulates well-characterised computational components involved in learning and decision-making. We found evidence that DBS specifically modulates outcome sensitivity during trial and error learning, which describes a propensity of action outcomes to modulate those same actions in the future. Outcome sensitivity, for both rewards and losses, is an essential feature of learning from experience, and was modulated in the absence of any other observable parameters in our data.

Behavioural symptoms such as impulsivity reflect a complex, multi-faceted set of motivational processes. There is now relatively strong evidence, supported by coherent computational theory, that the STN modulates strategic aspects of the decision process, notably the ability to inhibit early responding and allow time to accrue evidence to enable execution of better decisions^16^,^38^. In particular, this may reflect a general process that governs decision strategy when there is conflict in decision value^15^, decision system^12^ and perceptual evidence^39^.

However, whether choice *per se* is modulated, outside of the context of requirement to respond quickly as tested here, is much less clear^40^. Previous rodent studies suggested a role for STN in instrumental decision-making, including in a 5-choice food reward task^9^, and in inter-temporal choice for immediate versus delayed reward^41^. On the whole, this evidence suggests that lesions^42^,^43^,^44^, high frequency stimulation (HFS)^2^, or pharmacological blockage^45^ influence appetitive (reward) motivation. In humans, previous studies have suggested that STN-DBS impairs performance on a task which involves compound integration of previous trial-and-error learned outcome probabilities^46^, and results in a tendency towards risky decision-making in gambling tasks^47^. In both cases, however, there is context that invokes variable decision conflict. Furthermore, no previous study has, to our knowledge, shown a role for the STN in *punishment* related choice.

Our data implicate a relatively specific impact on value sensitivity over and above other aspects of decision-making, but only observed here in the context of learned and (bearing in mind it is difficult to formally contrast the two tasks) not explicit choice in discounting behaviour^48^). A tendency to choose small, sooner options in inter-temporal choice is often considered the most characteristic feature of behavioural impulsivity^49^. The lack of any positive evidence of temporal impulsivity is strengthened by a design in which subjects made real-life decisions for ecologically valid quantities and delays, and by the fact that small doses of the dopamine precursor levodopa can induce significant impulsivity, albeit in young, healthy individuals^29^. Thus, DBS may modulate learned value specifically, with no comparable effect on explicitly given or constructed outcome value. The only caveat to this is that the response times are not equitable between the two tasks: in the discounting task, the options are far more varied and the decision needs to made on the basis of new information on each trial, and hence subjects take much longer.

It is important to note that reduced instrumental outcome sensitivity cannot easily be attributed to a non-specific task-performance effect. For example, a general increase in switching behaviour^50^,^51^ (via the softmax temperature) would manifest as decreased sensitivity to reward and *increased* sensitivity to punishment – i.e. the effect on punishment being the opposite to what we observe here. Furthermore, in our discounting task, there is a tendency towards *more* consistent behaviour when DBS is ON. Instead, our data suggest a basic and specific reduction in outcome sensitivity during learning across rewards and punishments.

This raises a deeper question of what underlies this reduction in learned value, and how this relates to existing models of STN function in decision-making. One conceivable explanation is that such an effect could relate to a lower threshold of value-based choice computation, as predicted by decision difficulty theories^14^,^15^,^16^,^17^,^18^. That is, even though we imposed no explicit penalty for slowness, it may be that ON subjects internally integrate values too quickly (i.e. *decision time*), before the full option values have had time to accrue, but where this is not necessarily coupled to (relatively) faster *response time*. Such an effect would suggest a more pervasive effect on decision-making than previously thought, but not necessarily inconsistent with existing theories of STN function.

If the above is the case, it is notable that in a previous instrumental learning study^15^ in human PD subjects, STN DBS did not induce an overall deficit in the ability to select rewards or avoid punishments, in contrast to our findings. Instead, the authors’ task highlighted a selective increase in errors associated with conflict related speeding when ON stimulation. By comparison, our bandit task is much more dependent on flexible instrumental performance, and the combination of the non-stationarity of the option-outcome contingencies may make it more sensitive to value acquisition and variable option values. In addition, our analysis methodology allowed us to more clearly disambiguate effects of outcome value and learning. Indeed, our data support previous assertions that the STN does not appear to be involved in learning *per se*^15^,^52^. Furthermore, our data show similar effects on both reward and punishment value coding. This contrasts with a directional, valence-specific role, seen with dopaminergic medication (which similarly improves Parkinson’s disease symptoms), which accentuates reward-based decisions but *impairs* avoidance^53^,^54^.

An important caveat that should be noted is that the computational account of behaviour relies to a certain amount on the models applied. For instance, we haven’t considered more complex instantiations of reinforcement learning that consider multiple values systems that compete to control choices (i.e. model-based and model-free systems)^55^,^56^ or different classes of model, such as Bayesian decision algorithms^57^. Having said that, the reinforcement learning model does offer a parsimonious account that is broadly consistent with current conceptions of human decision-making.

In conclusion, our results show that both sensitivity to reward and punishments is reduced in the context of experience-based decision-making in DBS treated Parkinson’s disease. The results broaden our understanding of the subtle but important impact of STN-DBS on behaviour, in particular because it highlights an impact on learning and choice. This could result in clinical manifestations distinct from those that fall under the umbrella of impulsivity, but suggest other aspects of adaptive behaviour might be influenced and should be considered in future clinically-orientated evaluations of behaviour. Perhaps the best example would be in sensitivity to rehabilitation, since this involves experience-based learning from both positive and negative outcomes.

## Additional Information

**How to cite this article**: Seymour, B. *et al*. Deep brain stimulation of the subthalamic nucleus modulates sensitivity to decision outcome value in Parkinson’s disease. *Sci. Rep.* **6**, 32509; doi: 10.1038/srep32509 (2016).

## Figures and Tables

**Table 1:** Performance in terms of win and loss tokens accrued, and net income, when on and off stimulation.

	Wins	Losses	Net win
ON	€88.05 (5.84),	€80.23 (5.13).	€7.82 (3.06).
OFF	€93.64 (5.93),	€78.14 (4.46).	€15.50 (3.40)
			p < 0.05 sign test

SEM appears in parentheses.

**Table 2:** Frequency of repeating an option given previous outcome.

	All outcomes	Win	Loss	Win+Loss	No win/loss
ON	0.737 (0.034)	0.918 (0.023)	0.486 (0.043)	0.741 (0.043)	0.850 (0.044)
OFF	0.731 (0.033	0.944 (0.02)	0.374 (0.048)	0.739 (0.044)	0.867 (0.036)
Paired 2-tailed t-test	n/s	p = 0.08	p < 0.005	n/s	n/s

The table shows the frequency (expressed as a proportion) of choice repetition given the various outcomes it yielded on the preceding trial. ‘Win’ and ‘Loss’ outcomes refer to outcomes that yielded win or loss alone, and do not include simultaneous wins and losses. ‘Win+Loss’ refers to previous choices that yielded simultaneous win and loss tokens, and No win/loss refers to previous choices that yielded nothing. SEM are shown in parentheses.

**Table 3:** Instrumental learning task: parameter estimates from the model-based analysis (SEM in parentheses).

	Reward sensitivity	Reward learning rate	Loss sensitivity	Loss learning rate	Perseverative magnitude
ON	2.86 (0.42)	0.46 (0.07)	−1.85 (0.26)	0.63 (0.07)	2.40 (0.27)
OFF	4.46 (0.72)	0.42 (0.05)	−2.43 (0.39)	0.59 (0.06)	2.38 (0.31)
Paired t-contrast	p < 0.05	n/s	p < 0.05	n/s	n/s

**Table 4:** Normalised response times and performance on easy and hard trials when ON and OFF.

	RT Easy trials	RT hard trials	% correct easy trials	% correct hard trials
ON	0.489	0.519	82.6	60.3
OFF	0.459	0.500	89.3	58.8
Interaction	P = 0.08		n/s	

**Table 5:** Comparison of inter-temporal choice models.

Model	Number of parameters	Negative Log Likelihood	Bayesian Information Criteria
Hyperbolic discounting with linear utility	2	167.57	347.31
Exponential discounting with linear utility	2	164.29	340.75
**Hyperbolic discounting with power utility**	**3**	**145.34**	**308.94**
Hyperbolic discounting with negative exponential utility	3	154.22	326.70
Exponential discounting with power utility	3	156.38	331.02
Exponential discounting with negative exponential utility	3	149.85	317.96

The table illustrates the Likelihoods, complexity, and BIC scores for different neuro-economic models of inter-temporal choice. The best model, Hyperbolic discounting with power utility, is highlighted. Performing this analysis separately for both ON and OFF reveals a similar result (data not shown). The winning model corresponds to a model probability of about 0.72 on each trial.

**Table 6:** Parameter estimates of inter-temporal choice model (SEM in parentheses).

	k (hyperbolic discounting)	r (power utility function)	*τ* (temperature)
**ON**	**0.022 (0.007)**	**0.40 (0.07)**	**0.71 (0.39)**
**OFF**	**0.027 (0.007)**	**0.49 (0.08)**	**1.20 (0.58)**
Contrast paired two-sided t-test p-value (uncorrected)	0.758	0.178	0.049
Correlation coefficient with BIS score (r)	0.04	0.30	−0.04
Correlation with BIS score p-value	0.851	0.188	0.853

**Table 7:** Decision conflict.

	Low conflict trials	High conflict trials	Contrast p-value (two-sided paired t-test)
ON	0.5372	0.4970	0.0245
OFF	0.5418	0.4913	0.0048
Interaction			n/s (p = 0.364)

The table shows Gamma-normalised response latency as a function of decision conflict, which was defined by the difference between the value if the two options were presented on each trial.
